# Smart Home IoT Network Risk Assessment Using Bayesian Networks

**DOI:** 10.3390/e24050668

**Published:** 2022-05-10

**Authors:** Miguel Flores, Diego Heredia, Roberto Andrade, Mariam Ibrahim

**Affiliations:** 1 Grupo MODES, SIGTI, Facultad de Ciencias, Escuela Politécnica Nacional, Quito 170525, Ecuador; miguel.flores@epn.edu.ec; 2 Facultad de Ciencias, Escuela Politécnica Nacional, Quito 170525, Ecuador; diego.heredia01@epn.edu.ec; 3 Facultad de Ingeniera Sistemas, Escuela Politécnica Nacional, Quito 170525, Ecuador; 4 Department of Mechatronics Engineering, Faculty of Applied Technical Sciences, German Jordanian University, Amman 11180, Jordan; mariam.wajdi@gju.edu.jo

**Keywords:** Internet of Things (IoT), smart home, Bayesian network, simulation, risk assessment

## Abstract

A risk assessment model for a smart home Internet of Things (IoT) network is implemented using a Bayesian network. The directed acyclic graph of the Bayesian network is constructed from an attack graph that details the paths through which different attacks can occur in the IoT network. The parameters of the Bayesian network are estimated with the maximum likelihood method applied to a data set obtained from the simulation of attacks, in five simulation scenarios. For the risk assessment, inferences in the Bayesian network and the impact of the attacks are considered, focusing on DoS attacks, MitM attacks and both at the same time to the devices that allow the automation of the smart home and that are generally the ones that individually have lower levels of security.

## 1. Introduction

The Internet of Things (IoT) is an infrastructure that includes physical devices, modern vehicles, buildings and even essential electrical devices that we use daily, which are interconnected with each other through the Internet to accumulate and exchange data between them [1]. IoT solutions often use different types of topologies. One example are star topologies where IoT nodes are connected to a central hub that allows the exchange of messages between nodes for the execution of a specific function of the IoT system, such as turning on a light or closing a door [2]. However, the high interconnectivity between IoT devices is one of the factors that drives its application in different scenarios, although the IoT node is connected to a central device, which can also have a connection with other devices and systems based on a mesh topology [3]. This interconnectivity increases the possibilities of developing functionalities in IoT devices [4]. For example, turning on the light and closing the door can be achieved directly from the cell phone or using a voice assistant [5]. In this context, it is common to find a hybrid scheme in the use of these two types of topologies that handle a star topology with a hub or gateway to connect to other networks or manage their devices, as well as a mesh topology to allow communication between IoT devices. Some protocols used in the field of smart home that would fit with this approach include the following: Zigbee is an open standard for wireless technology of low cost and low power consumption, used for communication of devices, such as lights or plugs, in the ISM band with a range between 10 and 20 m [6]. Thread is IPv6-based wireless communication protocol for home automation that allows communication over power lines, radio frequency or a combination of both. Thread is also based in 6LoWPAN and it operates in the ISM band with range of 30 m [7]. Bluetooth Low Energy (BLE) operates in the ISM band and allows connectivity with tablets and smartphones. BLE support has range of coverage of 100 mts [8]. Finally, Z-Wave is a sub-1 GHz wireless mesh network specifically developed for smart home products, such as lights, door locks, security systems and heating control [9].

One of the key features of these technologies mentioned above is that they are wireless technologies with high interconnectivity. From a security perspective, the susceptibility to security attacks increases due to the use of shared channels. Although certain controls are established, for example, Z-wave generates a list of its devices and Thread introduces encryption, the security of IoT systems should also take control of devices that are connected through BLE. At this point, we can consider that these technologies use different authentication schemes, so we have the possibility of a third problem related to the heterogeneity of authentication mechanisms of IoT devices [10]. Finally, the high interconnectivity generates another problem related to the possibility of generating cascade effects in which the failure of a device affects the operability of other connected devices due to the interconnectivity between devices in mesh networks [11].

IoT networks are used in several fields such as health, transportation, education, agriculture, home automation and smart cities, among others. Each manufacturer establishes specific security measures for their devices; however, these security measures may not be entirely effective when the devices are interconnected in an IoT network. One of the most important problems is security related to cyber attack events on devices in the network. These events represent a risk for the people involved and can have a great economic impact.

Risk is defined through the probability of an undesirable event and its level of impact [12]. Jacobsson et al. [13] developed a risk analysis method for a smart home automation system based on this principle. The difference from the model presented in this work is that we focus on cyber attacks in the network and the relationships between them. In general, risk assessment models can be developed with the methodology of Bayesian networks [14]. In this context, risk assessment methodologies traditionally have a deterministic approach and some deterministic models that have been developed for the evaluation of cyber attacks are attack trees, Petri nets and attack graphs. For example, Sommestead [15] studied attack graph analysis tools in computer network security. Attack graph models allow for a better description of the network attack process, which is why they have become one of the most used tools for solving network security problems [16]. On the other hand, probabilistic models have also been studied. Wu et al. [17] developed a cyber attack prediction model based on Bayesian networks. Liu and Man [18] proposed a model of attack graphs based on Bayesian networks. Additionally, Frigault and Wang [19] developed a similar methodology for the study of network security. Improvements to the Liu and Man model were implemented; for example, Frigault et al. [20] added the factor of time to establish a dynamic model. In these models, the results presented as probabilities of attacks are obtained through inferences on the Bayesian networks. For this reason, algorithms have been developed to make the calculations efficient. Liu and Man used the variable elimination algorithm; however, it has low computational efficiency and it can only be used in small-scale networks [16]. Thus, Muñoz-González et al. [21] used the junction tree algorithm as an improvement of the variable elimination algorithm. Experiments showed that this algorithm is better than the variable elimination algorithm in terms of time complexity and spatial complexity and is more suitable for real situations [16]. The listed algorithms are known as exact algorithms and are feasible for Bayesian networks with few nodes. On the other hand, approximate algorithms for inferences have also been studied [22], and those are used for Bayesian networks with a larger number of nodes.

The risk assessment model implemented in this work is based on Bayesian network theory and the simulation of discrete random variables. The graph of the Bayesian net-work used in the model of this work is based on an attack graph of an IoT network of a smart home. This allows to represent the information of the possible paths that an attacker can follow to achieve their objective. Bayesian networks consider the causal relationship between random variables, and this is similar to attack graphs since, given an edge in an at-tack graph, the state of the destination node conditionally depends on the state of its source node; that is, the success of an attack conditionally depends on the success of an attack on the previous node [18]. One of the main advantages of using Bayesian networks for risk assessment in this type of network is the flexibility and ease of parameter training.

In the field of cybersecurity, the lack of data, especially historical data on breaches, incidents and threats, makes it difficult to develop realistic models; however, Bayesian networks have the potential to address this challenge [23]. Specifically, the ability to combine different sources of knowledge allows Bayesian networks to be a suitable model for the study of cybersecurity. The parameters of Bayesian networks can be estimated using data sets or expert opinion, but this information is scarce or of limited access to the public in the case of IoT networks. To solve this problem, in previous models using Bayesian networks, to determine the parameters, the Common Vulnerability Scoring System (CVSS) scores have been used [18,19,20,21], which are values that estimate the impact of the vulnerabilities of the devices in the networks. However, these scores do not consider the conditional dependency relationships at the nodes of the graph. In this context, due to the lack of information on attacks on IoT networks or the limitation of access to it, the need to implement mathematical simulation algorithms is considered, in order to obtain data sets that describe the attacks on the IoT network of a smart home and in this way approximate the parameters of the Bayesian network. Furthermore, by calculating inferences in the Bayesian network and using the simulated data, an adequate evaluation of the risks in the IoT network is obtained.

The rest of this paper is structured as follows. Section 2 shows the structure of smart home IoT networks and lists the cyber attacks that are considered for the model implementation. Section 3 shows the mathematical methods used for the model, which are Bayesian networks and simulation of Bernoulli random variables and vectors. Section 4 details the model implementation, including the construction of the Bayesian network, attack simulation and impact and risk calculation. Section 5 shows the principal results obtained from the model. Section 6 presents the discussion and Section 7 presents the conclusions of the project.

## 2. Smart Home IoT Networks

A smart home is a system made up of devices interconnected through an IoT network in a house. Some motivations for using IoT networks at home are safety, because a house can be monitored through security cameras and emergency calls can be automated, and power efficiency, because smart home devices can be turned on or off as needed or to save energy [24].

The structure of the IoT network in the smart home that we use in the model is presented in [25]:

- Access Point: The attacker is positioned at this point.
- Management Level: This level consists of devices that give orders such as laptops and cellphones (CP).
- Router: The router that all devices communicate with.
- Field Devices Level: At this level, all the devices related to the automation of the smart home are located, such as shutters, smart lights, air conditioner and smart cameras.

The smart home has three Wi-Fi connections between these levels [25]:

- Wi-Fi Network 1: Provides connection between access point and management level.
- Wi-Fi Network 2: Provides connection between management level and router.
- Wi-Fi Network 3: Provides connection between router and field devices level.

The following attacks are considered in the model implementation: social engineering (SE), phishing (PH), malware injection (MI), denial of service (DoS), routing table poisoning (RTP), persistent attack (PA) and man in the middle (MitM).

## 3. Methods

### 3.1. Bayesian Networks

A Bayesian network for a set of variables $X=\left\{X_{1},\ldots ,X_{n}\right\}$ is a pair (G, θ), where G is a directed acyclic graph with n nodes, one for each variable in X, called the network structure and θ is a set of conditional probability functions, called the network parametrization [26,27,28]. For each variable, its parameter is the set of conditional probability functions of the variable given each value of its parents in the graph. According to the type of random variable, three types of Bayesian networks are distinguished: discrete if the variables are discrete, continuous if the variables are continuous, and hybrid if there are both discrete and continuous variables in the network. In our model, a discrete Bayesian network containing Bernoulli random variables is considered.

The graph of a Bayesian network encodes a set of conditional independence assumptions, called local independencies: each variable $X_{i}$ is conditionally independent on its non-descendants given its parents in the graph.

An important result of Bayesian networks is the chain rule, which states that a Bayesian network for a set of variables $X=\left\{X_{1},\ldots ,X_{n}\right\}$ satisfies that

(1) $$P\left(X_{1},\ldots ,X_{n}\right)=\overset{n}{\underset{i=1}{\prod }}P(X_{i}|Pa\left(X_{i}\right))$$

where $Pa\left(X_{i}\right)$ are the parents of $X_{i}$ in the graph.

In Bayesian network theory, model selection and estimation are known as learning, and it consists of two steps: learning the structure of the Bayesian network, which is, the directed acyclic graph; and learning the parameters, which are the local probability distributions [28]. In this work, the directed acyclic graph will be obtained from the attack graph proposed in [25]. For parameter learning, we use the maximum likelihood method for a data set. This method states that given a data set of instantiations of the variables, in a discrete Bayesian network, each probability of the parameters is estimated with the formula:

(2) $$\theta_{x_{i}|pa\left(x_{i}\right)}=\frac{D\left(x_{i},pa\left(x_{i}\right)\right)}{D\left(pa\left(x_{i}\right)\right)}$$

where $D\left(x_{i},\;pa\left(x_{i}\right)\right)$ is the number of cases in the data set that contain the instantiation $x_{i}$ of $X_{i}$ and $pa\left(x_{i}\right)$ of its parents $Pa\left(X_{i}\right)$; and $D\left(pa\left(x_{i}\right)\right)$ is the number of cases in the data set that contain the instantiation $pa\left(x_{i}\right)$ of $Pa\left(X_{i}\right)$.

Once the Bayesian network has been designed, i.e., the structure and parameters have been specified, the objective is to obtain information by answering questions about the variables and their probabilities [26,28]. The techniques used are known in general as inference. For Bayesian networks, the process of answering these questions is also known as probabilistic reasoning or belief updating, while the questions themselves are called queries [28]. The queries used for risk assessment are the probabilities of evidence, posterior marginals, and most probable explanations. Considering that we use Bernoulli random variables in the model, given a Bayesian network for a set of Bernoulli random variables $X=\left\{X_{1},\ldots ,X_{n}\right\}$ these inferences are:

- Probability of evidence: $P\left(X_{i}=x_{i}\right)$ for $x_{i}\in \left\{0,1\right\},\;i\in \left\{1,\ldots ,n\right\}$.
- Posterior marginals: Let $X_{j}=x_{j}$, be evidence of the Bayesian network, posterior mar-ginals are $P\left(X_{i}=x_{i}│X_{j}=x_{j}\right)\;\forall \;x_{i}\in \left\{0,1\right\},\;\forall \;i\in \left\{1,\ldots ,j-1,j+1,\ldots ,n\right\}$.
- Most probable explanation: Let $X_{n}=x_{n}$, be evidence of the Bayesian network, most probable explanation are $x_{i}\in \left\{0,1\right\},\;\forall i\in \left\{1,\ldots ,n-1\right\}$ such that $P\left(X_{1}=x_{1},\;\ldots ,X_{n-1}=x_{n-1}│X_{n}=x_{n}\right)$ is maximum.

The algorithms for the calculation of inferences are classified as exact and approximate. Exact algorithms are based on the application of Bayes’ theorem and due to their nature, they are feasible for small networks. On the other hand, approximate algorithms use Monte Carlo simulations to sample the global distribution of the network and thus estimate inferences, and those algorithms are useful in Bayesian networks with a large number of nodes. In this work, an exact algorithm is used due to the precision of the results and its feasibility because the Bayesian network graph of our model has few nodes. Specifically, the junction tree algorithm is used.

### 3.2. Simulation

To simulate Bernoulli random variables and vectors, we use the Inverse Transform Method [29]. For the simulation of a single Bernoulli variable with parameter *p*, we generate a random number *U*, uniformly distributed over the interval (0,1) and set:

- A value of 0 if U<p;
- A value of 1 if U≥p.

For the simulation of a random vector with two Bernoulli variables with parameters $p_{ij}$, we generate a random number *U*, uniformly distributed over the interval (0,1) and set:

- (0,0) if $U<p_{00}$;
- (0,1) if $p_{00}\leq \;U<p_{00}+p_{01}$;
- (1,0) if $p_{00}+p_{01}\leq \;U<p_{00}+p_{01}+p_{10}$; or
- (1,1) if $U\geq p_{00}+p_{01}+p_{10}$.

Finally, for the simulation of a random vector with three Bernoulli variables with parameters $p_{ijk}$, we generate a random number *U*, uniformly distributed over the interval (0,1) and set:

- (0,0,0) if $U<p_{000}$;
- (0,0,1) if $p_{000}\leq U<p_{000}+p_{001}$;
- (0,1,0) if $p_{000}+p_{001}\leq U<p_{000}+p_{001}+p_{010}$;
- (0,1,1) if $p_{000}+p_{001}+p_{010}\leq U<p_{000}+p_{001}+p_{010}+p_{011}$;
- (1,0,0) if $p_{000}+p_{001}+p_{010}+p_{011}\leq U<p_{000}+p_{001}+p_{010}+p_{011}+p_{100}$;
- (1,0,1) if $p_{000}+p_{001}+p_{010}+p_{011}+p_{100}\leq U<p_{000}+p_{001}+p_{010}+p_{011}+p_{100}+p_{101};$
- (1,1,0) if $p_{000}+p_{001}+p_{010}+p_{011}+p_{100}+p_{101}\leq U<p_{000}+p_{001}+p_{010}+p_{011}+p_{100}+p_{101}+p_{110}$; or
- (1,1,1) if $U\geq p_{000}+p_{001}+p_{010}+p_{011}+p_{100}+p_{101}+p_{110}$.

## 4. Model Implementation

In this section, we present the model to assess the risk of the smart home IoT network. We explain how to obtain the structure of the Bayesian network, the attack simulation algorithm, the parameters of the Bayesian network and finally, the calculation of impact and risk.

### 4.1. Bayesian Network Structure

#### 4.1.1. Attack Graph

**Figure 1:**
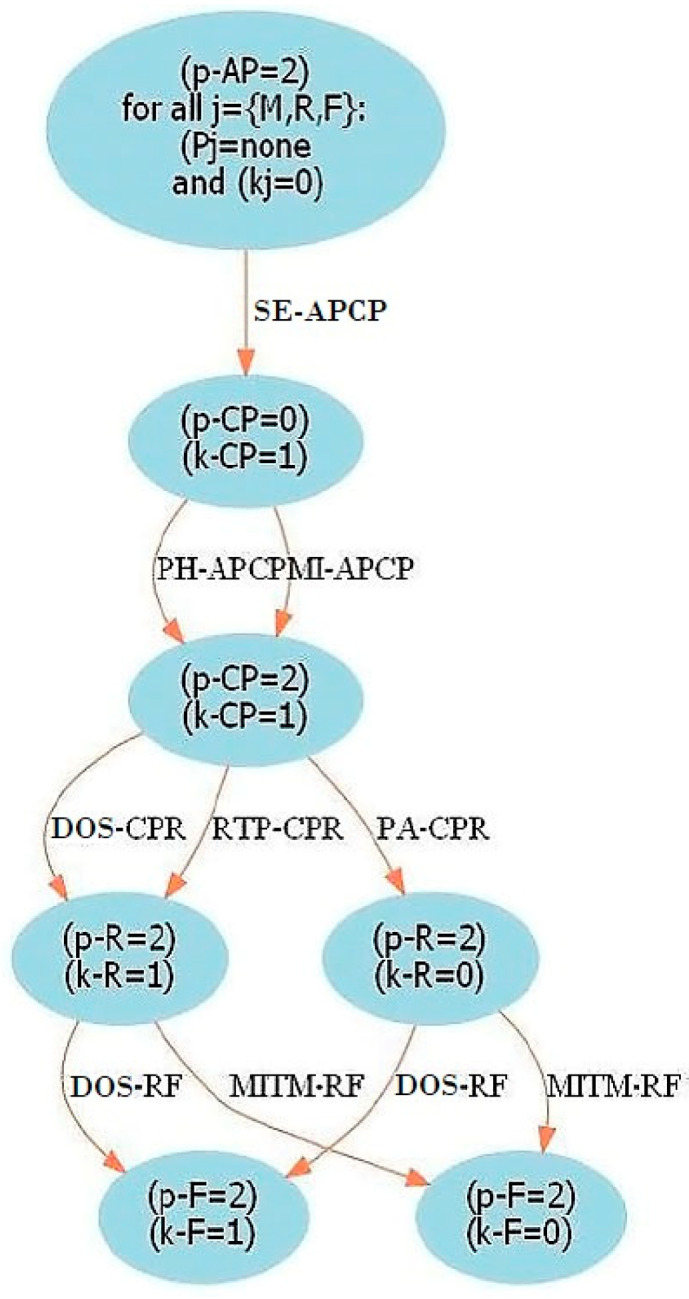
Smart home IoT network attack graph. Elaborated by Ibrahim and Nabulsi [25].

An attack graph is a graphical model that represents the knowledge about the vulnerabilities in a network, showing the different paths that an attacker can follow to reach a certain objective. In the smart home IoT network, an attack graph describes the order in which the different attacks on the devices must occur in order to reach another device that is considered as a target. The attack graph of the smart home IoT network that is used for the model was developed by Ibrahim and Nabulsi [25] and is shown in Figure 1. This graph is based on the structure of the smart home IoT network, i.e., the access point, management level, router and field devices level, as well as the cyber attacks mentioned in Section 2. The target in the attack graph is the field devices level, so the paths describe the ways in which it can be attacked. The attack graph model proposed by Ibrahim and Nabulsi [25] analyzes the ways in which different attacks can occur in the smart home IoT network. On the other hand, the model developed in this work starts from the attack graph to design the directed acyclic graph that forms the structure of a Bayesian network. The Bayesian network is then used to determine the probability of occurrence of attacks, their impact and their risk, complementing the study of cybersecurity in smart homes.

In the attack graph shown in Figure 1, nodes represent the state of each level of the IoT network structure in terms of information (k), where 0 means that the attacker has no information and 1 means that they do; and the type of privileges the attacker has (p), where 0 means no privileges, 1—user privileges, and 2—root privileges. The edges represent the type of attack and the devices involved. In this way, in the graph, 12 possible paths can be distinguished in which the attacker manages to obtain root privileges at the field devices level. The levels of the network structure are denoted as follows: access point (AP), administration level (M), however, only one cell phone (CP) is considered to be at this level, router (R) and the field devices level (F). Attacks are denoted as follows: social engineering (SE), phishing (PH), malware injection (MI), denial of service (DOS), routing table poisoning (RTP), persistent attack (PA) and man in the middle (MITM). In this way, for each node, the state p and k of the network level are placed with their respective value. Each edge of the graph represents an attack, the level of origin and the level of destination of this attack; for example, SE-APCP means that a social engineering attack can occur from the access point to the management level.

The model developed in this work uses the attack graph to design the directed acyclic graph of a Bayesian network. The Bayesian network is then used to determine the probability of attacks to the field devices level, their impact and risk.

#### 4.1.2. Directed Acyclic Graph

The set of variables of the Bayesian network of the model is based on the attack graph presented by Ibrahim and Nabulsi. We consider discrete random variables with Bernoulli distribution since they are used to determine the state of the attacks in the network. The variables take the value of 1 if the attack occurs and 0 otherwise. The following Bernoulli random variables are considered for the implementation of the Bayesian network:

- SECP: A social engineering attack is performed from the access point to the management level, which consists of a cellphone.
- PHCP: A phishing attack is performed from the access point to the management level.
- MICP: A malware injection attack is performed from the access point to the management level.
- DOSR: A DoS attack is performed from management level to the router.
- RTPR: A routing table poisoning attack is performed from management level to the router.
- PAR: A persistent attack is performed from management level to the router.
- DOSF: A DoS attack is performed from the router to the field devices level.
- MITMF: A MitM attack is performed from the router to the field devices level.

**Figure 2:**
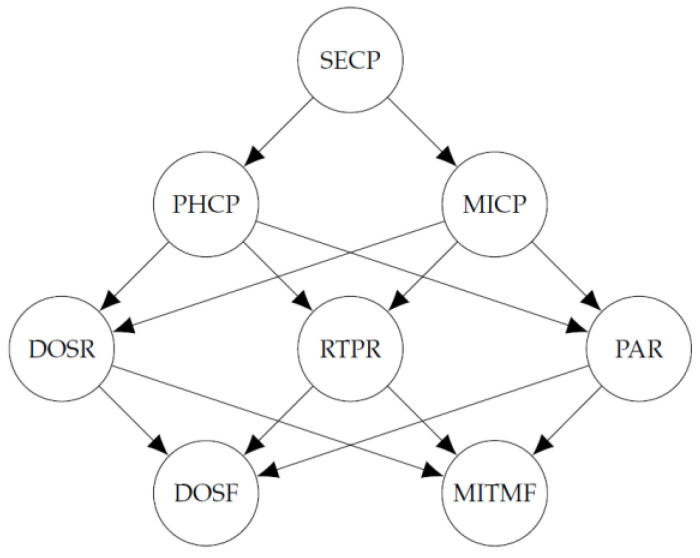
Directed acyclic graph of the Bayesian network.

Considering the smart home IoT network structure, the attack graph presented in [25] and the variables mentioned above, we use the directed acyclic graph of Figure 2 for the implementation of the Bayesian network. In this graph, all the paths in which an attack can be performed to the field devices level are represented.

The directed acyclic graph of Figure 2 was obtained considering all the paths through which attacks occur in the IoT network, which are shown in Figure 1. It should be noted that the attack graph considers the field devices level as the target, therefore, attack paths are directed at this level of the network. In the directed acyclic graph, each node represents the occurrence of an attack between devices at different levels of the IoT network structure. For example, in the attack graph, the DOS-RF edge indicates a DoS attack from the router to the field devices level and in the directed acyclic graph, this is represented by the DOSF node. Thus, with the definition of the nodes and the edges of the directed acyclic graph, the same paths of the attack graph are represented.

### 4.2. Attack Simulation

The objective of the simulation is to describe how cyber attacks are performed through the nodes of the network and this is achieved by simulating discrete random variables. First, the attacks that are performed at each level of the smart home IoT network structure are selected and then it is determined whether the attacker has managed to compromise the devices. As a result of the simulation, a data set is obtained where each column represents a network node and each row represents a complete attack event on the network, indicating the nodes through which the attacks were made. General considerations for simulation are that the attacker can perform multiple attacks from one node and attacks that do not follow the paths described by the graph are not allowed.

#### 4.2.1. Attack Selection

For each level of the smart home IoT network structure, the first step of the simulation is to decide the attack or attacks to be performed and this is done by simulating random vectors of 2 or 3 Bernoulli variables. These variables take the value of 1 if the node is selected and 0 otherwise. It is considered that the attacker always chooses at least one of the available attacks at each level, therefore, the first node of the graph (SECP) will always be chosen, and it is not necessary to simulate its selection. The vectors to simulate are:

- Attack selection at the administration level: $\left(S_{PHCP},\;S_{MICP}\right)$ where $S_{PHCP}$—a phishing attack is selected and $S_{MICP}$—a malware attack is selected.
- Attack selection at the router level: $\left(S_{DOSR},\;S_{RTPR},\;S_{PAR}\right)$ where $S_{DOSR}$—a DoS attack is selected, $S_{RTPR}$—an RTP attack is selected and $S_{PAR}$: a persistent attack is selected.
- Attack selection at field devices level: $\left(S_{DOSF},\;S_{MITMF}\right)$ where $S_{DOSF}$—a DoS attack is selected and $S_{MITMF}$—a MitM attack is selected.

#### 4.2.2. Simulation Scenarios

In this work, the risk assessment is focused on DoS and MitM attacks at the field devices level. The objective of proposing simulation scenarios is to compare different behaviors of the attackers in relation to the attacks that are selected at each level of the IoT network. This selection depends on the priority of protection that the smart home owner or risk assessor provides for the attacks on devices at each level of the IoT network. In this work, in the first scenario, the attacks are considered to have the same priority; in the second and fourth scenarios, higher priority is given to DoS attacks, which are aimed at blocking devices; and in the third and fifth scenarios, higher priority is given to MitM attacks whose goal is to intercept communication channels between devices. The values for the different simulation scenarios were determined as an example for the application of the methodology. However, depending on the needs or requirements of the network owner, any scenario can be implemented, giving it the priorities suggested by said owner. The parameters of the random vectors proposed for the selection of attacks are the probabilities of the joint probability function, and are called selection probabilities; these define the simulation scenarios and for each level of the IoT network, they are shown in Table 1, Table 2 and Table 3.

When the vectors take the values (0,0), (0,0,0) and (0,0), no attacks are selected and in that case the probability of 0 is assigned because for the model we consider that the attacker always chooses at least one attack at each level of the IoT network structure.

At the administration and field devices levels, when the vector is (1,1), a probability of 0.10 is assigned for all scenarios because attackers generally choose only one type of attack, although the possibility of a double attack is not discarded and, therefore, a low probability of 0.10 is considered. At the router level, when the vector is (1,1,1), a probability of 0.04 is considered for all scenarios, since it is not common for an attacker to choose three different types of attacks simultaneously for a single device, however, this possibility is not discarded.

For the rest of values, the description is the following:

- Scenario 1: At the administration and field devices level, for simple attacks, i.e., when the vectors are (0,1) and (1,0), an equal probability of 0.45 is considered. At the router level, a probability of 0.25 is considered for simple attacks, i.e., (0,0,1), (0,1,0) and (1,0,0); and a probability of 0.07 is considered for double attacks, i.e., (0,1,1), (1,0,1) and (1,1,0).
- Scenarios 2 and 4: For both scenarios, the effect of assigning a higher probability to $S_{MICP}$ than to $S_{PHCP}$ is analyzed at the management level. At the router level, for simple attacks, the order from highest to lowest probability is considered as follows: $S_{DOSR}$, $S_{RTPR}$ and $S_{PAR}$. For double attacks, the order from highest to lowest probability is considered as follows: $S_{DOSR}$ and $S_{RTPR}$, then $S_{DOSR}$ and $S_{PAR}$, and finally, $S_{RTPR}$ and $S_{PAR}$. For the field devices level, $S_{DOSF}$ is considered to have higher probability than $S_{MITMF}$. The difference between Scenario 4 and Scenario 2 is that in the second scenario, a medium incidence is assigned and in the fourth, a high incidence is assigned to the attacks with the highest probability at each level.
- Scenarios 3 and 5: For both scenarios, the effect of assigning a higher probability to $S_{PHCP}$ than to $S_{MICP}$ is analyzed at the management level. At the router level, for simple attacks, the order from highest to lowest probability is considered as follows: $S_{RTPR}$, $S_{PAR}$ and $S_{DOSR}$; and for double attacks: $S_{RTPR}$ and $S_{PAR}$, then $S_{DOSR}$ and $S_{RTPR}$ and finally $S_{DOSR}$ and $S_{PAR}$. For the field devices level, $S_{MITMF}$ is considered to have higher probability than $S_{DOSF}$. The difference between Scenario 3 and Scenario 5 is that in the third scenario, a medium incidence is assigned and in the fifth, a high incidence is assigned to the attacks with the highest probability at each level.

#### 4.2.3. Vulnerabilities and CVSS Scores

The Common Vulnerability Scoring System (CVSS) is a scoring system that measures the impact or severity of vulnerabilities in Information Technology security. CVSS scores are based on the principal technical characteristics of software, hardware and firmware vulnerabilities [30]. In general, three metrics are used to calculate these scores: base, temporal and environmental. The base score reflects the severity of a vulnerability according to its intrinsic characteristics that are constant over time, and is estimated based on the worst case in different scenarios [30]. Temporal metrics are described based on factors that change over time and environmental metrics are described according to a specific environment [30].

CVSS scores have been used in previous works to estimate the parameters of discrete Bayesian networks in risk assessment models for computer systems [16,18,19,20,21]. In [20,21], it is considered that the probability of exploiting a vulnerability is the CVSS score divided for the size of the domain, which is 10. However, this approach does not consider the causal relationships between the attacks present in the graph. In this work, the CVSS scores are not used as parameters of the Bayesian network, but as one of the criteria for the simulation of attacks. The base metrics are transformed to probabilities following the approach of [20], i.e., dividing the base CVSS score by 10, and this value is used as the parameter of a Bernoulli random variable. Then, a value of this random variable is simulated and the result indicates whether the attacker was successful in performing the attack. These variables are called vulnerability variables and are the second criterion for the attack simulation. For each variable in the Bayesian network, we use the following notation: at the administration level—$V_{SECP}$, $V_{PHCP}$ and $V_{MICP}$, at the router level—$V_{DOSR}$, $V_{RTPR}$ and $V_{PAR}$, and at the field devices level—$V_{DOSF}$ and $V_{MITMF}$. The base metrics used for the calculation of these scores are Attack Vector (AV), Attack Complexity (AC), User Interaction (UI), Privileges Required (PR), Scope (S), Confidentiality (C), Integrity (I) and Availability (A). A full description of the values of these metrics can be found in [30], and an official CVSS score calculator is provided in [31].

In Table 3, the values of these metrics applied to our variables, the base CVSS score and the parameter of each variable are displayed.

The metrics that are used in Table 4 to determine the vulnerability probabilities are the following:

- Attack Vector (AV): Indicates the context in which the exploitation of vulnerabilities is possible. The base score is higher the more remote the attack is. The values it can take are: Network (N), Adjacent (A), Local (L) and Physical (P).
- Attack Complexity (AC): Describes the conditions beyond the attacker’s control that must be in place to complete the attack. The base score is higher for less complex attacks. The values it can take are: Low (L) and High (H).
- User Interaction (UI): Determines whether the vulnerability can be exploited solely at the will of the attacker, or whether a user must participate in some way. The base score is higher when no user interaction is required. The values it can take are: None (N) and Required (R).
- Privileges Required (PR): Describes the privileges an attacker must have before exploiting a vulnerability. The base score is higher if no privileges are required. The values it can take are: None (N), Low (L) and High (H).
- Scope (S): Determines if a vulnerability affects resources in components beyond its security scope. The base score is higher if the scope is modified. The values it can take are: Changed (C) and Unchanged (U).
- Confidentiality (C): Measures the impact on the confidentiality of information due to a successfully exploited vulnerability. Confidentiality refers to limiting access and disclosure of information to authorized users only, as well as preventing access or disclosure to unauthorized persons. The base score is higher when the loss of the affected component is higher. The values it can take are: None (N), Low (L) and High (H).
- Integrity (I): Measures the integrity impact of a successfully exploited vulnerability. Integrity refers to the reliability and truthfulness of information. It mainly refers to the modification of data and the type of control that the attacker has in the modification of the information. The base score is higher when the consequence for the affected component is higher. The values it can take are: None (N), Low (L) and High (H).
- Availability (A): Measures the impact on the availability of the affected component as a result of a successfully exploited vulnerability. This metric refers to the loss of availability of the affected component itself, such as a network service (web, database, email). The base score is higher when the consequence for the affected component is higher. The values it can take are: None (N), Low (L) and High (H).

#### 4.2.4. Simulation Algorithm

Considering the selection and vulnerability variables defined in Section 4.2.1 and Section 4.2.3, Algorithm 1 is developed to simulate network attacks.
**Algorithm 1.** Attack Simulation.$\mathrm{Input}:\;N,S_{PHCP},S_{MICP},S_{DOSR},S_{RTPR},S_{PAR},S_{DOSF},S_{MITMF},V_{SECP},V_{PHCP},V_{MICP},V_{DOSR},V_{RTPR},\;V_{PAR},V_{DOSF},V_{MITMF}$Output: Attack data set XFor n=1 to n=N, do$\;\;\mathrm{Simulate}\;V_{SECP}$$\;\;\mathrm{If}\;V_{SECP}=1,\;\mathrm{then}$$\;\;\;\;\mathrm{Simulate}\;\left(S_{PHCP},\;S_{MICP}\right)$$\;\;\;\;\mathrm{For}\;i\in \left\{P_{HCP},M_{ICP}\right\}\;\mathrm{such}\;\mathrm{that}\;S_{i}=1\;\mathrm{in}\;\mathrm{the}\;\mathrm{previous}\;\mathrm{step},\;\mathrm{simulate}\;V_{i}$$\;\;\;\;X_{n,1}=\;\{i\in \left\{PHCP,MICP\right\}|V_{i}=1\;\mathrm{in}\;\mathrm{the}\;\mathrm{previous}\;\mathrm{step}\}$$\;\;\;\;\mathrm{If}\;\left|X_{n,1}\right|\neq 0,\;\mathrm{then}$$\;\;\;\;\;\;\mathrm{For}\;j\in X_{n,1},\;\mathrm{do}$$\;\;\;\;\;\;\;\;\mathrm{Simulate}\;\left(S_{DOSR},\;S_{RTPR},\;S_{PAR}\right)$$\;\;\;\;\;\;\;\;\mathrm{For}\;i\in \left\{DOSR,RTPR,PAR\right\}\;\mathrm{such}\;\mathrm{that}\;S_{i}=1\;\mathrm{in}\;\mathrm{the}\;\mathrm{previous}\;\mathrm{step},\;\mathrm{simulate}\;V_{i}$$\;\;\;\;\;\;\;\;X_{n,2,j}=\left\{i\in \left\{DOSR,RTPR,PAR\right\}|V_{i}=1\;\mathrm{in}\;\mathrm{the}\;\mathrm{previous}\;\mathrm{step}\right\}$$\;\;\;\;\;\;X_{n,2}=\underset{j\in X_{n,1}}{\cup }X_{n,2,j}$$\;\;\;\;\;\;\mathrm{Remove}\;\mathrm{repeated}\;\mathrm{elements}\;\mathrm{of}\;X_{n,2}$$\;\;\;\;\;\;\mathrm{If}\;\left|X_{n,2}\right|\neq 0,\;\mathrm{then}$$\;\;\;\;\;\;\;\;\mathrm{For}\;k\in X_{n,2}\;\mathrm{do}$$\;\;\;\;\;\;\;\;\;\;\mathrm{Simulate}\;\left(S_{DOSF},\;S_{MITMF}\right)$$\;\;\;\;\;\;\;\;\;\;\mathrm{For}\;i\in \left\{DOSF,MITMF\right\}\;\mathrm{such}\;\mathrm{that}\;S_{i}=1\;\mathrm{in}\;\mathrm{the}\;\mathrm{previous}\;\mathrm{step},\;\mathrm{simulate}\;V_{i}$$\;\;\;\;\;\;\;\;\;\;X_{n,3,k}=\;\{i\in \left\{DOSF,MITMF\right\}|V_{i}=1\;\mathrm{in}\;\mathrm{the}\;\mathrm{previous}\;\mathrm{step}\}$$\;\;\;\;\;\;\;\;X_{n,3}=\underset{k\in X_{n,2}}{\cup }X_{n,3,k}$$\;\;\;\;\;\;\;\;\mathrm{Remove}\;\mathrm{repeated}\;\mathrm{elements}\;\mathrm{of}\;X_{n,3}$$\;\;\;\;\;\;\;\;X_{n}=\left\{SECP\right\}\cup^{\;}X_{n,1}\cup^{\;}X_{n,2}\cup^{\;}X_{n,3}$      Else$\;\;\;\;\;\;\;\;X_{n}=\left\{SECP\right\}\cup^{\;}X_{n,1}$    Else$\;\;\;\;\;\;X_{n}=\left\{SECP\right\}$  Else$\;\;\;\;X_{n}=\emptyset$$X=\overset{N}{\underset{n=1}{\cup }}X_{n}$


### 4.3. Bayesian Network Parametrization

Once the structure of the Bayesian network has been designed and the attack data sets have been obtained through simulation, the parameters of the Bayesian network are estimated using the maximum likelihood method. The following parameters are calculated:

- $\theta_{SECP}$;
- $\theta_{PHCP|SECP}$;
- $\theta_{MICP|SECP}$;
- $\theta_{DOSR|PHCP,MICP}$;
- $\theta_{RTPR|PHCP,MICP}$;
- $\theta_{PAR|PHCP,MICP}$;
- $\theta_{DOSF|DOSR,RTPR,PAR}$; and
- $\theta_{MITMF|DOSR,RTPR,PAR}$.

The parameters of the Bayesian network represent the set of conditional probability functions of each node given its parents in the graph. In this case, since the variables are discrete, each probability function is a set of probabilities. Table 4 presents the parameters of the proposed Bayesian network and the set of probabilities that are estimated for each parameter.

### 4.4. Inferences for Risk Assessment

Considering that the objective of the attack graph is to prevent attacks at the field devices level [25], the risk assessment is based on this principle and the following inferences are computed on the Bayesian network:

- Probabilities of evidence: P(DOSF=1), P(MITMF=1) and P(DOSF=1, MITMF=1).
- Posterior marginals: Considering Y=1 as evidence, with Y∈{SECP,PHCP,MICP, DOSR,RTPR,PAR}; P(DOSF=1|Y=1), P(MITMF=1|Y=1), and P(DOSF=1,MITMF=1|Y=1) is calculated. P(DOSF=1|PHCP,MICP), P(MITMF=1|PHCP,MICP), P(DOSF=1,MITMF=1|PHCP,MICP) is also calculated for all values of PHCP and MICP.
- Most probable explanations: For the DoS attack, the values of SECP, PHCP, MICP, DOSR, RTPR and PAR are determined, such that: P(SECP,PHCP,MICP,DOSR, RTPR, PAR|DOSF=1,MITMF=0) is maximum. For the MitM attack, the values of SECP, PHCP, MICP, DOSR, RTPR and PAR are determined, such that: P(SECP,PHCP,MICP, DOSR,RTPR,PAR|DOSF=0,MITMF=1) is maximum.

### 4.5. Attack Impact

In this work, the impact is considered as the loss of confidentiality, integrity and availability in the devices if they are successfully compromised by any attack. Thus, the proposed method for calculating the impact is based on the CVSS scores and includes some base, temporal and environmental metrics [32]. The base metrics used are Confidentiality (C), Integrity (I), and Availability (A). The environmental metrics are Confidentiality Requirement (CR), Integrity Requirement (IR) and Availability Requirement (AR). These metrics customize the CVSS score according to the importance of the affected asset, measured in terms of confidentiality, integrity and availability. The temporal metric used is the Remediation Level (RL) and this refers to the type of solutions that are proposed for the vulnerabilities. The numeric values for each metric are described in [32] and the impact is calculated with the formula:

(3) $$Impact=\frac{CR\times R+IR\times I+AR\times A}{3}\times \left(1-RL\right)$$

Impact is represented as a score from 0 to 100, where 0 indicates no impact and 100—maximum impact. Table 5 shows the values of the metrics considered for each node, along with the impact value.

The base metrics used in Table 6 are Confidentiality (C), Integrity (I) and Availability (A) which were explained for vulnerability variables. The environmental metrics are Confidentiality Requirement (CR), Integrity Requirement (IR) and Availability Requirement (CA), and they allow the CVSS score to be customized according to the importance of the affected asset, measured in terms of confidentiality, integrity and availability. These metrics can take the following values: High (H) when the loss of confidentiality, integrity, or availability is likely to have a catastrophic adverse effect on the organization or people associated with the organization (for example, employees, customers); Medium (M) when the adverse effect is serious; Low (L) when the adverse effect is limited; and Not Defined (N) when there is not enough information to choose one of the other values. The temporal metric used is the remediation level (RL) and this refers to the type of solutions that are proposed for the vulnerabilities. Typically, device vulnerabilities are not fixed as soon as they are discovered, and workarounds are provided until an official one is issued. Each of the steps up to the official solution reduces the temporary CVSS score and in this case, the impact value. The less official and permanent a fix is, the higher the impact score is. The values it can take are: Not Defined (X), Unavailable (U), Workaround (W), Temporary Fix (T) and Official Fix (OF).

In the data sets obtained by simulation, to determine the impact of a complete attack on the network, which is a row of the dataset, the impact values of the nodes in which the attacks occurred are added. To calculate the impact of an event that includes a specific attack, the rows where the attack occurred on the target node are filtered and the mean of the sum of the impacts of these rows is obtained. In the case of conditional probabilities, the rows where the value of the variable which is to be evaluated and the evidence occurred are filtered and the mean is obtained in the same way.

### 4.6. Risk Assessment

In this work, the risk is considered as a function of the probability of an attack on a device and the impact that this attack generates. The risk of events is calculated with the formula [32]:

(4) Risk=Probability×Impact

The probabilities of events are obtained from the inferences in the Bayesian network that were explained in Section 4.4 and the impact is obtained from the method described in Section 4.5.

Individually, the attacks on each device, i.e., each node, can have an impact value between 0 and 100. On the other hand, to calculate the impact of a network attack event, all the impact values of the nodes for which the attack occurred are added together. Since the graph has eight nodes, the impact of an event can take values between 0 and 800. Considering that the impact on each node is fixed, then the highest impact value that an event can take is 423.67, which is the sum of the values in the Impact column of Table 5. Considering that the probabilities can take values between 0 and 1 and that the risk is calculated with Formula (4), then the risk can only take values between 0 and 423.67.

The risk value that is calculated for different attack events is used for comparative purposes to determine the type of attack that represents greater or lesser risk at the field devices level; and its comparison with the double attack of DoS and MitM.

## 5. Results

In this section we present the results of the risk assessment in the smart home IoT network, analyzing the attacks at the field devices level. For each simulation scenario, 10,000 attack data points were simulated.

**Figure 3:**
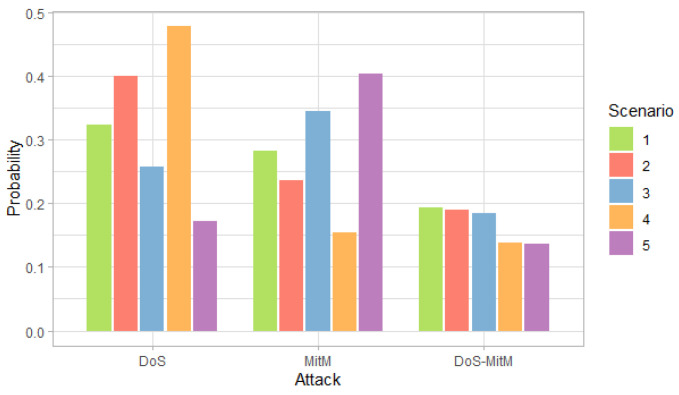
Probability of attacks at the field devices level by scenarios.

**Figure 4:**
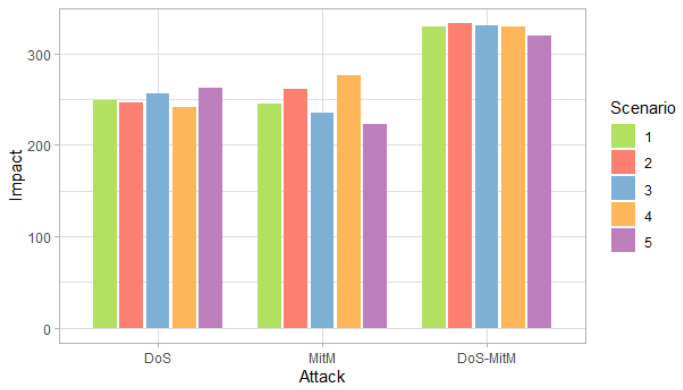
Impact of attacks at the field devices level by scenarios.

**Figure 5:**
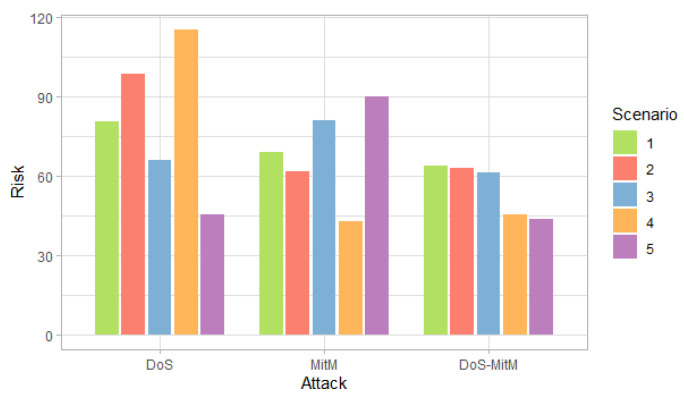
Risk of attacks at the field devices level by scenarios.

Figure 3, Figure 4 and Figure 5 show the probability, impact and risk of DoS, MitM and both DoS and MitM attacks at the same time at the field devices level, without considering evidence of attacks on the other nodes.

Figure 3 shows that, for the first, second and fourth scenarios, the DoS attack is more likely than the MitM attack, while the opposite occurs for the third and fifth scenarios. On the other hand, for all scenarios, the probability of any of the two simple attacks is greater than the probability of the double attack.

Figure 4 shows that the impact for DoS and MitM attacks is similar in all scenarios. On the other hand, the double attack has a greater impact than the simple attacks.

Figure 5 shows that the DoS attack has a higher risk in the first, second and fourth scenarios and the MitM attack in the third and fifth scenarios, following the trend of the probabilities of Figure 3. Although the impact of the double attack is greater than the impact of simple attacks, the risk of a double attack is lower than the risk of simple attacks in most scenarios because risk is defined as the product of probability and impact.

**Figure 6:**
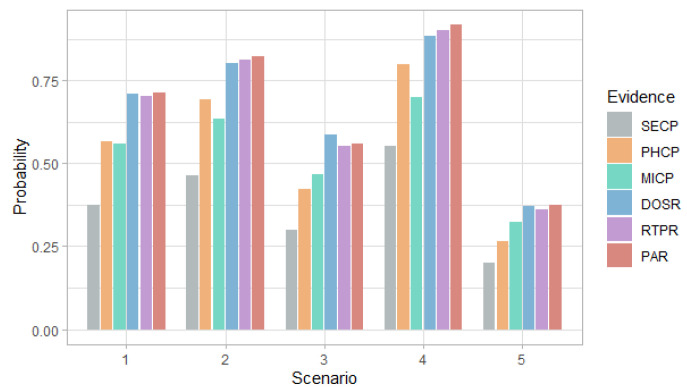
Probability of DoS attack at the field devices level in each scenario considering the evidence of attack on the other nodes.

**Figure 7:**
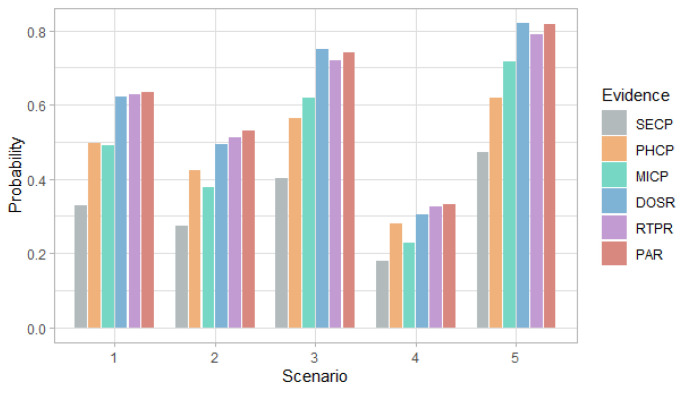
Probability of MitM attack at the field devices level in each scenario considering the evidence of attack on the other nodes.

**Figure 8:**
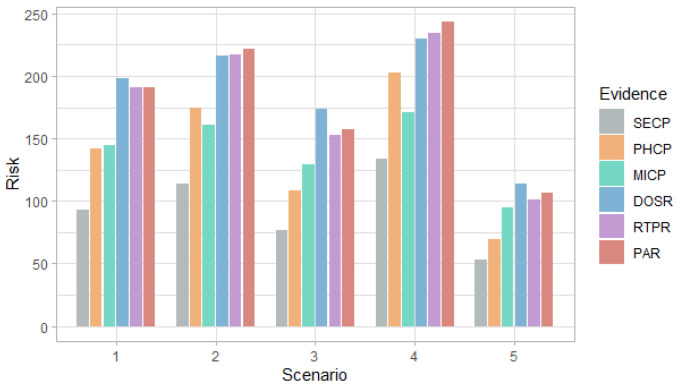
Risk of DoS attack at the field devices level in each scenario considering the evidence of attack on the other nodes.

**Figure 9:**
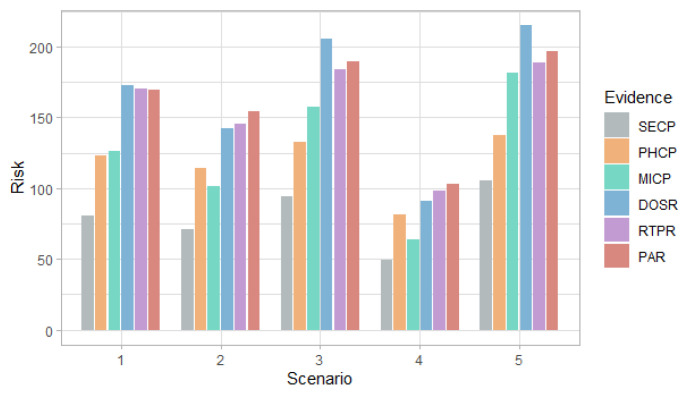
Risk of MitM attack at the field devices level in each scenario considering the evidence of attack on the other nodes.

Figure 6, Figure 7, Figure 8 and Figure 9 show the probability and risk of DoS and MitM attacks in the field devices level considering one variable as evidence in the Bayesian network, specifically, the evidence of an attack at the management and router levels.

Figure 6 and Figure 7 show that in the first scenario, despite the fact that the selection probabilities are the same, the DoS attack has a higher probability than the MitM attack, with any of the evidence. This behavior also occurs in the case of the risk in Figure 8 and Figure 9.

In the second and fourth scenarios, both the probability and the risk are higher for the DoS attack, compared to the MitM attack; and for the third and fifth scenarios, they are higher for the MitM attack. This behavior is expected due to the assignment of the selection probabilities for the definition of the simulation scenarios. However, the benefit of this analysis is that the values can be quantified. For example, in Figure 8, it can be seen that when there is evidence of attacks at the router level (DOSR, RTPR and PAR), the DoS attack at the field devices level has risks greater than 200 in the second and fourth scenarios; whereas, in Figure 9, it can be seen that for the MitM attack, the risk only exceeds the value of 200 when the DOSR attack evidence is given in the third and fifth scenarios.

Now, in order to analyze the effect of increasing evidence in the Bayesian network, we consider that two nodes in the graph are attacked. The expected behavior is that the values of probability, impact and risk increase for all scenarios, with respect to the case when only one type of attack is considered to occur as evidence and when no attacks are considered as evidence.

**Figure 10:**
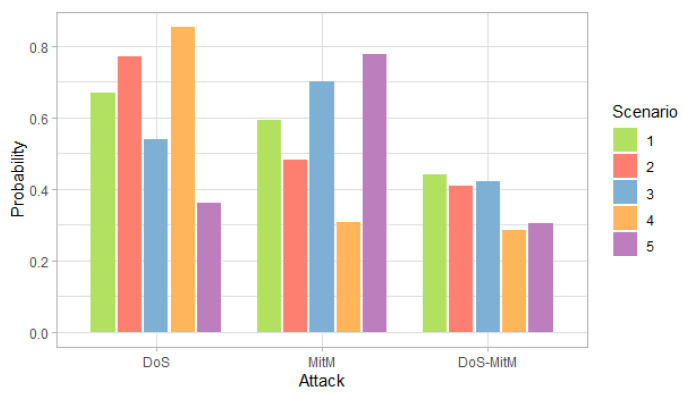
Probability of attack at the field devices level considering the evidence of phishing and malware attacks at the administration level, by scenario.

**Figure 11:**
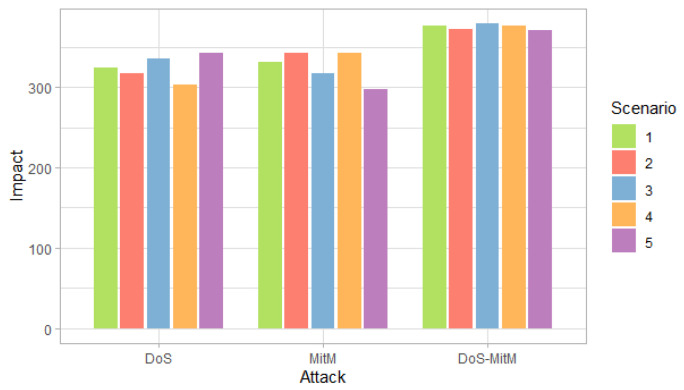
Impact of attack at the field devices level considering the evidence of phishing and malware attacks at the administration level, by scenario.

**Figure 12:**
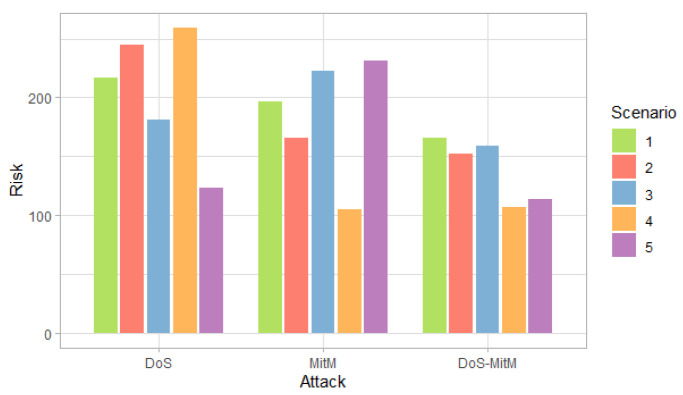
Risk of attack at the field devices level considering the evidence of phishing and malware attacks at the administration level, by scenario.

Figure 10, Figure 11 and Figure 12 show the probability, impact and risk of DoS, MitM and double DoS and MitM attacks at the field devices level considering as evidence that phishing and malware attacks have occurred at the management level (PHCP and MICP).

Figure 10 shows that for the first, second and fourth scenarios, the DoS attack is more likely than the MitM attack, while the opposite occurs in the rest of the scenarios. For all scenarios, the probability of simple attacks is higher than double attacks. This behavior is the same as observed in Figure 3, however, in this case, the probabilities are higher for all scenarios because there is evidence that two attacks occurred.

Figure 11 shows that the impact values for simple attacks are similar and the values for the double attack are greater than the simple ones, for all scenarios. In addition, only in the fourth scenario for the DoS attack and in the fifth for the MitM attack, the impact value is less than 300, which shows an increase in the values with respect to the case in which no evidence is given, presented in Figure 4.

Figure 12 shows that the DoS attack predominates in the first, second and fourth scenarios, and the MitM attack—in the remaining scenarios. In addition, the highest risk for each scenario is greater than 200, which shows an increase compared to when there was evidence of phishing or malware attack at the administration level, which can be seen in Figure 8 and Figure 9.

The mentioned figures present the results in three situations: without evidence of attacks, assuming as evidence that an attack occurred on any other of the nodes that are not the field devices level, and assuming as evidence that two attacks occurred at the management level. These study cases can be set according to the preferences of the risk assessor or smart homeowner. The cases considered for this model were determined considering what could be situations of greater interest. On the other hand, any evidence about the model can be considered and the desired probability calculated. It should be noted that in this model, the results focus on the field devices level because it is the objective to be protected in the IoT network.

Considering the chain rule for Bayesian networks and the variables in the model presented, specific probabilities that include the specification of all variables in the model can be calculated with the formula:

$$\begin{array}{l} P\left(SECP,PHCP,MICP,DOSR,RTPR,PAR,DOSF,MITMF\right)\\ \;\;\;\;\;\;\;=P\left(SECP\right)\times P\left(PHCP|SECP\right)\times P\left(MICP|SECP\right)\times P\left(DOSR|PHCP,MICP\right)\times P\left(RTPR|PHCP,MICP\right)\\ \;\;\;\;\;\;\;\times P\left(PAR|PHCP,MICP\right)\times P\left(DOSF|DOSR,RTPR,PAR\right)\times P\left(MITMF|DOSR,RTPR,PAR\right) \end{array}$$

It is worth mentioning that all the probabilities on the right side of the equation are the parameters of the Bayesian network that we estimate with the maximum likelihood method.

Now, considering the most probable explanations inferences in the Bayesian network, we set as evidence that there was an attack at the field devices level and look for the nodes of the administration and router levels that are attacked such that the probability given the evidence is the maximum.

**Figure 13:**
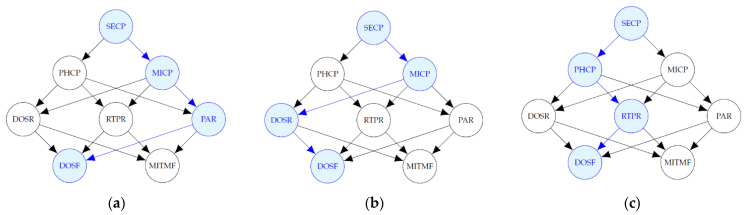
Most probable explanations for DOSF. (**a**) Most probable explanation for DOSF in Scenario 1; (**b**) Most probable explanation for DOSF in Scenarios 2 and 4; (**c**) Most probable explanation for DOSF in Scenarios 3 and 5.

Figure 13 includes diagrams of the most probable explanation, in each simulation scenario, when there is evidence that a DoS attack has occurred at the field devices level and a MitM attack has not occurred. The blue-colored nodes, except DOSF, represent the variables where the attacks have occurred that are not evidence, i.e., the variables that take value of 1 such that P(SECP,PHCP,MICP, DOSR,RTPR,PAR|DOSF=1,MITMF=0) is the maximum.

In this analysis, the path that maximizes the probability when there is evidence of a DoS attack, in the second and fourth scenarios, includes the malware attack at the administration level and DoS at the router level; and in the third and fifth scenarios it includes a phishing and RTP attack. This behavior was expected due to the assignment of the selection probabilities, however in the first scenario, in which these probabilities are the same for simple attacks, the path includes a persistent attack at the router level.

**Figure 14:**
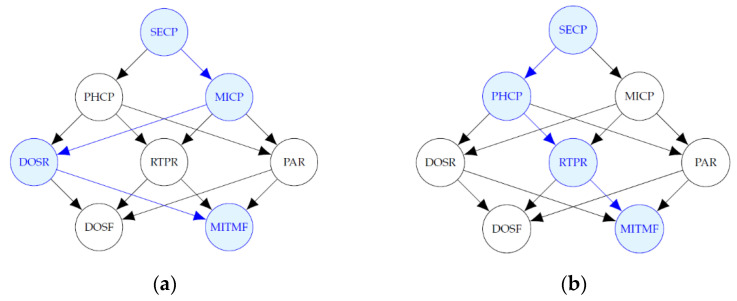
Most probable explanations for MITMF. (**a**) Most probable explanation for MITMF in Scenarios 1, 2 and 4; (**b**) Most probable explanation for MITMF in Scenarios 3 and 5.

Figure 14 shows diagrams of the most probable explanation, in each simulation scenario, when there is evidence that a MitM attack occurred at the field devices level and a DoS attack did not occur. The blue-colored nodes, except MITMF, represent the variables where the attacks occurred that are not evidence, i.e., the variables that take value of 1 such that P(SECP,PHCP,MICP, DOSR,RTPR,PAR|DOSF=0,MITMF=1) is the maximum.

The path that maximizes the probability when there is evidence of a MitM attack, in the first, second and fourth scenarios, includes the malware attack at the administration level and DoS at the router level; in the third and fifth scenarios, it includes a phishing and RTP attack. In this case, the first scenario is the same as the second and fourth; however, the behavior was expected for the other scenarios.

In general, it can be stated that the nodes in the most probable paths follow the trend that is assigned in the selection probabilities when defining the simulation scenarios. In the first scenario, since there is no preference for any of the attacks, any attack can be in the most probable explanation. Despite this, the malware attack coincided in both cases.

## 6. Discussion

The number of devices forms the attack surface of the IoT system. An increase in the number of devices would increase the attack surface and therefore the probability of success of the attacks by having a greater number of attackable devices. In the attack simulation process, as the number of nodes increases, new Bernoulli random vectors for attack selection and new Bernoulli random variables for the vulnerability of devices must be considered. New impact values should also be considered for each new node, which would increase the impact range of the events, which depends on the sum of the impact values of all nodes. Considering that the risk is the multiplication of the probability and the impact, then the increase in the number of nodes would increase the range of the risk value.

If new communication channels are considered through which the same or different attacks can occur, then the attack graph must first be modified so that all these new attack possibilities are represented. With the modified attack graph, Bernoulli random variables are defined as in the presented model (each variable represents the occurrence of an attack from one level to another of the IoT network) and placed in an acyclic directed graph, which forms the structure of a Bayesian network. On the other hand, for the parameters of the Bayesian network, the same process is followed with respect to the simulation and the estimation with the maximum likelihood method.

The probabilities selected for the scenario simulation are based on input from experts. These may vary according to the preferences of the risk assessor, the owner of the IoT network or the group of experts selected. At this point, techniques such as the fuzzy Delphi could be applied to handle subjectivity in this type of procedure. The contribution of this work focuses on the proposal to establish a methodology that allows risk assessment using a Bayesian model, and we consider that the probability values for the simulation probabilities were previously validated. The main idea is that, in the first scenario, the attacker has no preference in individual attacks; in the second and fourth scenarios, it has a preference over malware and DoS attacks, with a higher incidence in the fourth scenario; and finally, in the third and fifth scenarios, a preference over phishing, RTP and MitM attacks, with a higher incidence in the fifth scenario.

The methodology described in this work can be applied to any type of smart home IoT network. The most important thing is to be able to describe the attacks that can occur, organize them in an attack graph and determine both the probabilities for the random variables that are used for attack simulation and the metrics for calculating the impact of each attack.

The proposed risk assessment model is focused on setting a device or a level of the smart home IoT network structure as a target and representing all possible attacks at this target in a directed acyclic graph. The nodes of this graph represent the attacks between devices and the edges—the order of these attacks. In our case, the field devices level of the smart home IoT network is established as the target. On the other hand, in an IoT network with a mesh topology, there may be a greater number of paths through which devices can be attacked and this structure may represent the whole set of paths through which attacks can be performed to every device on the network. In order to apply the proposed methodology in this type of networks, a target device must also be considered, and a directed acyclic graph must be designed only with the attacks at this target. This analysis would therefore be detailed and specific to this node. Then, if required, a similar procedure could be performed for the other devices, depending on the risk assessor’s or owner’s preference.

Since there are differences in the risks of cyber attacks between continents such as America and Europe, a comparison in the risk assessment for both continents could be proposed as a possible future work. This type of analysis is not performed in this work due to the lack of information on attacks. The objective of the presented model is to propose a methodology that can be applied to future projects such as the comparison of both continents. The process to follow would be to build different attack graphs with the possible attacks and consider the probabilities according to each case. Then, simulations can be run or one can use real data and compare the results.

## 7. Conclusions

According to the analysis without considering evidence, the DoS attack represents the greatest risk for the field devices level, as it predominates in the largest number of scenarios. Thus, the greatest risk is that the attacker blocks the devices and prevents them from accessing the network. From the analysis, when one attack is considered as evidence and when two attacks are considered as evidence, the results in terms of trends are similar, which means that the DoS attack predominates in most cases.

The behavior of the attacks in terms of higher probability and risk follows what is assigned in the selection probabilities for the definition of the simulation scenarios for the second, third, fourth and fifth scenarios; however, in the first scenario, despite the fact that there is no preference for any attack, the DoS attack predominates in all the results. Although the double attack has a greater impact values, in relation to the risk, it is always lower than the simple attacks. This happens because risk is a function of both probability and impact of attacks.

The benefit of this type of analysis is the quantification of the risk values of different attacks. This allows for a more detailed analysis, which can lead to better decision making as to which attack should be given the highest priority for implementing security measures in the network. From the point of view of risk, in terms of the design of the smart home IoT network, more attention should be paid to DoS attacks than to MitM attacks at the field devices level, without neglecting attacks at the administration and router levels, since when there is evidence of attacks at these levels, a degree of incidence in the risk of attacks at the field devices level is observed.

Although the results show that the highest risk is found in the DoS attack, the needs of the owner of the network also influence in prioritizing risk. For this risk assessment, new simulation scenarios can be established such that a specific analysis can be carried out according to the requirements of the owner of the smart home IoT network.

## Figures and Tables

**Table 1:** Selection probabilities for the management level.

Variables		Scenarios				
$S_{PHCP}$	$S_{MICP}$	1	2	3	4	5
0	0	0.00	0.00	0.00	0.00	0.00
0	1	0.45	0.60	0.30	0.75	0.15
1	0	0.45	0.30	0.60	0.15	0.75
1	1	0.10	0.10	0.10	0.10	0.10

**Table 2:** Selection probabilities for the router level.

Variables			Scenarios				
$S_{DOSR}$	$S_{RTPR}$	$S_{PAR}$	1	2	3	4	5
0	0	0	0.00	0.00	0.00	0.00	0.00
0	0	1	0.25	0.20	0.25	0.15	0.25
0	1	0	0.25	0.25	0.30	0.25	0.35
0	1	1	0.07	0.06	0.08	0.05	0.09
1	0	0	0.25	0.30	0.20	0.35	0.15
1	0	1	0.07	0.07	0.06	0.07	0.05
1	1	0	0.07	0.08	0.07	0.09	0.07
1	1	1	0.04	0.04	0.04	0.04	0.04

**Table 3:** Selection probabilities for the field devices level.

Variables		Scenarios				
$S_{DOSF}$	$S_{MITMF}$	1	2	3	4	5
0	0	0.00	0.00	0.00	0.00	0.00
0	1	0.45	0.30	0.60	0.15	0.75
1	0	0.45	0.60	0.30	0.75	0.15
1	1	0.10	0.10	0.10	0.10	0.10

**Table 4:** CVSS base metrics and $V_{Node}$ variable parameters.

Node	AV	AC	UI	PR	S	C	I	A	CVSS	Parameter
SECP	N	L	N	N	C	H	N	N	8.6	0.86
PHCP	N	L	R	N	C	H	L	N	8.2	0.82
MICP	N	L	R	N	C	H	L	L	8.8	0.88
DOSR	N	L	N	H	C	H	L	H	9.0	0.90
RTPR	N	L	N	H	C	H	L	H	9.0	0.90
PAR	N	L	N	H	C	H	L	H	9.0	0.90
DOSF	N	H	N	N	C	H	H	H	9.0	0.90
MITMF	A	H	R	L	C	H	H	H	7.6	0.76

**Table 5:** Parameters of the Bayesian network.

Parameters	Probabilities
$\theta_{SECP}$	P(SECP)
$\theta_{PHCP|SECP}$	P(PHCP|SECP)
$\theta_{MICP|SECP}$	P(MICP|SECP)
$\theta_{DOSR|PHCP,MICP}$	P(DOSR|PHCP, MICP)
$\theta_{RTPR|PHCP,MICP}$	P(RTPR|PHCP, MICP)
$\theta_{PAR|PHCP,MICP}$	P(PAR|PHCP, MICP)
$\theta_{DOSF|DOSR,RTPR,PAR}$	P(DOSF|DOSR, RTPR, PAR)
$\theta_{MITMF|DOSR,RTPR,PAR}$	P(MITMF|DOSR, RTPR, PAR)

**Table 6:** Metrics for calculating the impact on the model.

Node	C	I	A	CR	IR	AR	RL	Impact
SECP	H	N	N	M	L	L	U	20.00
PHCP	H	L	N	M	L	L	OF	21.25
MICP	H	L	L	M	M	M	OF	34.00
DOSR	H	L	H	H	M	H	W	72.83
RTPR	H	L	H	H	M	M	W	60.17
PAR	H	L	H	H	L	M	W	55.42
DOSF	H	H	H	H	M	H	U	86.67
MITMF	H	H	H	M	M	H	U	73.33

## References

[1] Rajab H., Cinkelr T. IoT based smart cities. Proceedings of the 2018 International Symposium on Networks, Computers and Communications (ISNCC).

[2] Oliva S.L., Palmieri A., Invidia L., Patrono L., Rametta P. Rapid Prototyping of a Star Topology Network based on Bluetooth Low Energy Technology. Proceedings of the 2018 3rd International Conference on Smart and Sustainable Technologies (SpliTech).

[3] Taştan S.İ., Dalkiliç G. Smart Home System Using Internet of Things Devices and Mesh Topology. Proceedings of the 2021 6th International Conference on Computer Science and Engineering (UBMK).

[4] Johnson D., Ketel M. IoT: The Interconnection of Smart Cities. Proceedings of the 2019 SoutheastCon.

[5] Kaiborta A.K., Samal S. IoT based Voice Assistant for Home Automation. Proceedings of the 2022 4th International Conference on Smart Systems and Inventive Technology (ICSSIT).

[6] Gavra V.-D., Po O.A.P. Usage of ZigBee and LoRa wireless technologies in IoT systems. Proceedings of the 2020 IEEE 26th International Symposium for Design and Technology in Electronic Packaging (SIITME).

[7] Unwala I., Taqvi Z., Lu J. Thread: An IoT Protocol. Proceedings of the 2018 IEEE Green Technologies Conference (GreenTech).

[8] Boualouache A.E., Nouali O., Moussaoui S., Derder A. A BLE-based data collection system for IoT. Proceedings of the 2015 First International Conference on New Technologies of Information and Communication (NTIC).

[9] Varol A.B. Compilation of Data Link Protocols: Bluetooth Low Energy (BLE), ZigBee and Z-Wave. Proceedings of the 2019 4th International Conference on Computer Science and Engineering (UBMK).

[10] Unwala I., Taqvi Z., Lu J. IoT Security: ZWave and Thread. Proceedings of the 2018 IEEE Green Technologies Conference (GreenTech).

[11] Albalawi A., Almrshed A., Badhib A., Alshehri S. A Survey on Authentication Techniques for the Internet of Things. Proceedings of the 2019 International Conference on Computer and Information Sciences (ICCIS).

[12] Kandasamy K., Srinivas S., Achuthan K., Rangan V.P. (2020). IoT cyber risk: A holistic analysis of cyber risk assessment frameworks, risk vectors, and risk ranking process. EURASIP J. Inf. Secur..

[13] Jacobsson A., Boldt M., Carlsson B. (2016). A risk analysis of a smart home automation system. Future Gener. Comput. Syst..

[14] Kabir S., Papadopoulos Y. (2019). Applications of bayesian networks and petri nets in safety, reliability, and risk assessments: A review. Saf. Sci..

[15] Sommestad T., Sandström F. (2015). An empirical test of the accuracy of an attack graph analysis tool. Inf. Comput. Secur..

[16] Zeng J., Wu S., Chen Y., Zeng R., Wu C. (2019). Survey of attack graph analysis methods from the perspective of data and knowledge processing. Secur. Commun. Netw..

[17] Wu J., Yin L., Guo Y. Cyber attacks prediction model based on bayesian network. Proceedings of the 2012 IEEE 18th International Conference on Parallel and Distributed Systems.

[18] Liu Y., Man H., Dasarathy B.V. (2005). Network vulnerability assessment using Bayesian networks. Data Mining, Intrusion Detection, Information Assurance, and Data Networks Security 2005.

[19] Frigault M., Wang L. Measuring network security using bayesian network-based attack graphs. Proceedings of the 2008 32nd Annual IEEE International Computer Software and Applications Conference.

[20] Frigault M., Wang L., Singhal A., Jajodia S. (2008). Measuring network security using dynamic bayesian network. Proceedings of the 4th ACM Workshop on Quality of Protection, QoP’ 08.

[21] Muñoz-González L., Sgandurra D., Barrère M., Lupu E.C. (2019). Exact inference techniques for the analysis of bayesian attack graphs. IEEE Trans. Dependable Secur. Comput..

[22] Muñoz-González L., Sgandurra D., Paudice A., Lupu E.C. (2017). Efficient attack graph analysis through approximate inference. ACM Trans. Priv. Secur..

[23] Chockalingam S., Pieters W., Teixeira A., van Gelder P., Lipmaa H., Mitrokotsa A., Matulevicius R. (2017). Bayesian Network Models in Cyber Security: A Systematic Review; Secure IT Systems.

[24] Bastos D., Shackleton M., El-Moussa F. (2018). Internet of things: A survey of technologies and security risks in smart home and city environments. Living in the Internet of Things: Cybersecurity of the IoT—2018.

[25] Ibrahim M., Nabulsi I. Security analysis of smart home systems applying attack graph. Proceedings of the 2021 Fifth World Conference on Smart Trends in Systems Security and Sustainability (WorldS4).

[26] Darwiche A. (2009). Modeling and Reasoning with Bayesian Networks.

[27] Koller D., Friedman N. (2009). Adaptive Computation and Machine Learning series. Probabilistic Graphical Models: Principles and Techniquesi.

[28] Scutari M., Denis J.-B. (2021). Bayesian Networks: With Examples in R.

[29] Ross S. (2013). Simulation.

[30] Common Vulnerability Scoring System Version 3.1 Specification Document. https://www.first.org/cvss/v3.1/specification-document.

[31] Common Vulnerability Scoring System Version 3.1 Calculator. https://www.first.org/cvss/calculator/3.1.

[32] Aksu M.U., Dilek M.H., Tatli E.I., Bicakci K., Dirik H.I., Demirezen M.U., Aykir T. A quantitative CVSS-based cyber security risk assessment methodology for IT systems. Proceedings of the 2017 International Carnahan Conference on Security Technology (ICCST).

